# Self-Alignment of Bottom CZTSSe by Patterning of an Al_2_O_3_ Intermediate Layer

**DOI:** 10.3390/nano10010043

**Published:** 2019-12-23

**Authors:** Sanghun Hong, Se-Yun Kim, Dae-Ho Son, Seung-Hyun Kim, Young-Ill Kim, Kee-Jeong Yang, Young-Woo Heo, Jin-Kyu Kang, Dae-Hwan Kim

**Affiliations:** 1 School of Materials Science and Engineering, Kyungpook National University, Daegu 41566, Korea; shhong@dgist.ac.kr (S.H.); ywheo@knu.ac.kr (Y.-W.H.); 2 Research Center for Thin Film Solar Cells, Daegu-Gyeongbuk Institute of Science and Technology (DGIST), Daegu 42988, Korea; kimseyun@dgist.ac.kr (S.-Y.K.); dhson@dgist.ac.kr (D.-H.S.); seunghyun@dgist.ac.kr (S.-H.K.); lynx012@dgist.ac.kr (Y.-I.K.); kjyang@dgist.ac.kr (K.-J.Y.); 3 Division of Energy Technology, Daegu-Gyeongbuk Institute of Science and Technology (DGIST), Daegu 42988, Korea

**Keywords:** CZTSSe, intermediate layer, self-alignment, wettability, metal precursor, two-step

## Abstract

When CZTSSe is synthesized using a metal precursor, large voids of nonuniform size form at Mo back contact side. Herein, we demonstrate that the voids and CZTSSe in the lower part of the CZTSSe double layer can be controlled by using an Al_2_O_3_-patterned Mo substrate. The CZTSSe in the lower part self-aligns on the Mo-exposed area, while the voids self-align on the Al_2_O_3_-coated area. The origin of the self-alignment is expected to be the difference in bonding characteristics between liquid Sn and the metal or oxide surface, e.g., Al_2_O_3_. Good wettability generally forms between nonreactive liquid metals and metal surfaces due to the strong metallic bonding. By contrast, poor wettability generally forms between nonreactive liquid metals and oxide surfaces due to the weak van der Waals bonding between the liquid metal and the oxide layer. When the patterning was added, the device efficiency tended to decrease from 8.6% to 10.5%.

## 1. Introduction

Thin-film photovoltaic (PV) technology, such as CdTe and CIGSsolar cells, has been well developed and has opened up the possibility of integrating solar modules into buildings [1]. However, the issues of the Cd and Te toxicity and the scarcity of In, Ga, and Te remain for this technology. Therefore, the development of alternative thin-film PV technologies using earth-abundant and nontoxic materials is still a necessity. Hence, kesterite (Cu_2_SnZnS_4_, Cu_2_SnZnSe_4_, and Cu_2_ZnSn(S_1-x_Se_x_)_4_) thin-film solar cells are considered to be promising candidates for large-area module production [2,3]. To date, the highest power conversion efficiency of a CZTSSe cell has been reported as 12.6%; these cells were produced using the hydrazine solution process, which requires careful management to prevent explosions [4]. Thus, many attempts have been made to replace the hydrazine solution process [5].

Our group has been developing two-step processes using metal precursor stacks and chalcogen reactants. A 12.3% power conversion efficiency was reported for CZTSSe solar cells produced using the two-step process with a metal precursor and Se/SeS_2_ powder [6]. Recently, conversion efficiencies of 13.04% certified by KIER, a reliable certification institute in Korea, and of 12.62% certified by Newport PV Lab [3], one of the designated test centers participating in international round robins, were obtained for separate devices using Se powder and H_2_S gas as chalcogen reactants.

The formation mechanisms for the CZTSSe double layer, the ZnSSe layer within the CZTSSe double layer, and the large voids at the Mo back contact side, which have commonly been observed in CZTSSe when using a metal precursor, were explained in our previous work [7]; the formation of the ZnSSe shell by dezincification and the preferential reaction and mass transport of Cu and Sn can cause void formation. Additionally, the distribution of the bottom CZTSSe has been reported to depend on the surface properties of the substrate [8]; the bottom CZTSSe was observed to be relatively agglomerated on a soda-lime glass (SLG) or Al_2_O_3_-coated Mo/SLG substrate compared to the formation on a bare Mo/SLG substrate.

Based on the previous results, the ability to arrange the bottom CZTSSe or voids using an Al_2_O_3_-patterned Mo-SLG substrate was expected. Herein, we confirmed that the distribution of voids and the bottom CZTSSe can be controlled by intermediate layer patterning. The bottom CZTSSe was well aligned on the Mo-exposed area. The Al_2_O_3_ layer was deposited by RF magnetron sputtering, and the Al_2_O_3_-patterned Mo/SLG substrate was prepared by photolithography. The Sn/Cu/Zn metal precursor was deposited by DC magnetron sputtering, and then, a sulfo-selenization process was conducted using rapid thermal processing (RTP).

## 2. Experimental Details

A 600 nm-thick Mo layer was deposited on an SLG substrate via DC magnetron sputtering using a Mo target with a 99.99% purity. A 130 nm-thick Al_2_O_3_ layer was deposited by RF magnetron sputtering, and the Al_2_O_3_ pattern was prepared by conventional photolithography and wet etching using buffered oxide etchant (BOE) solution. Afterward, the metal precursors for the CZTSSe absorber layer were deposited on the Al_2_O_3_-patterned Mo layer using 99.99% pure Sn, Cu, and Zn sputtering targets with a stacking order of Sn/Cu/Zn/Mo. H_2_S (250 sccm) and Ar (2000 sccm) gas were supplied until the chamber pressure reached 700 Torr. The sample was heated at 300 °C for 15 min and then the sample was heated to 480 °C for 10 min. 50 nm-CdS buffer layer was·oated by chemical bath deposition. The 50 nm-thick intrinsic ZnO layer and 300 nm-thick Al-doped ZnO layer were sequentially deposited by RF-sputtering. Finally, a 2 µm-thick Al grid was deposited using e-beam evaporation. The experimental details regarding the sulfo-selenization process were given in previous work [7]. The samples were characterized by scanning transmission electron microscopy-energy dispersive spectrometry (STEM-EDS). STEM-EDS (Bruker Co., model QUANTAX-200) measurements were performed to analyze the compositional map of the samples.

## 3. Results and Discussion

**Figure 1:**
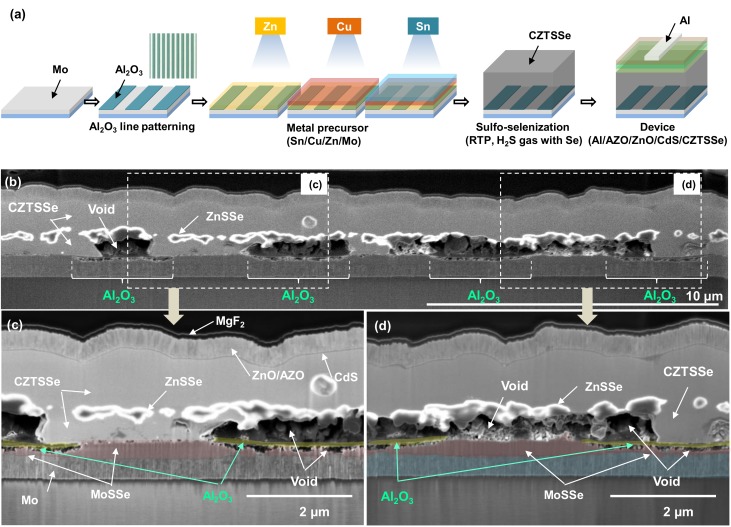
(**a**) Scheme of the CZTSSe fabrication process using an Al_2_O_3_-patterned Mo-SLG substrate. (**b**) Cross-sectional FESEM image of the self-aligned CZTSSe layer; cross-sectional views were obtained after FIB sampling (Ga ion milling). (**c**,**d**) Magnified FESEM images of the parts shown in Figure 1b.

As shown in Figure 1a, the line-patterned Al_2_O_3_ was prepared by photolithography and wet etching. The widths of the lines and spacings are 4 µm and 3 µm, respectively. Zn, Cu and Sn were sequentially deposited on the Al_2_O_3_-patterned Mo-SLG substrate. The CZTSSe film was synthesized by a sulfo-selenization reaction using RTP. Then, CdS was coated using the chemical bath deposition (CBD) method, ZnO and AZO were coated using the sputtering method, and Al electrodes were coated by thermal evaporation.

Figure 1b shows a cross-sectional field-emission scanning electron microscopy (FESEM) image of a self-aligned CZTS film. To repeatedly view the Al_2_O_3_-covered and Mo-exposed areas, the samples were cut perpendicular to the Al_2_O_3_ line direction. The previous report described well that when a sulfo-selenization reaction occurs while using an Sn/Cu/Zn metal stack, a CZTSSe double layer, a ZnSSe layer within the CZTSSe double layer, and large voids at the Mo back contact side form [7]. Regardless of Al_2_O_3_ patterning, the top CZTSSe layer is dense and can be seen as a continuous film, and a ZnSSe layer exists between the top CZTSSe and bottom CZTSSe layers, as shown in Figure 1b. Interestingly, when an Al_2_O_3_-patterned Mo-SLG substrate is used, in many cases, voids form in the areas coated with Al_2_O_3_, while the bottom CZTSSe is located where the Mo is exposed, as shown in Figure 1b,c. However, as shown in Figure 1b,d. the bottom CZTSSe is partially present on the Al_2_O_3_ film, and voids are also partially present at the exposed locations. Thick MoSSe is observed at the exposed locations, while thin MoSSe is found under the Al_2_O_3_ film, as shown in Figure 1c,d. Below the Al_2_O_3_ passivation layer, a thin MoSSe layer and fine voids are observed. This result seems to occur because the sputtered Al_2_O_3_ passivation layer does not effectively block chalcogen source diffusion; thus, the MoSSe layer is formed below the Al_2_O_3_ layer.

**Figure 2:**
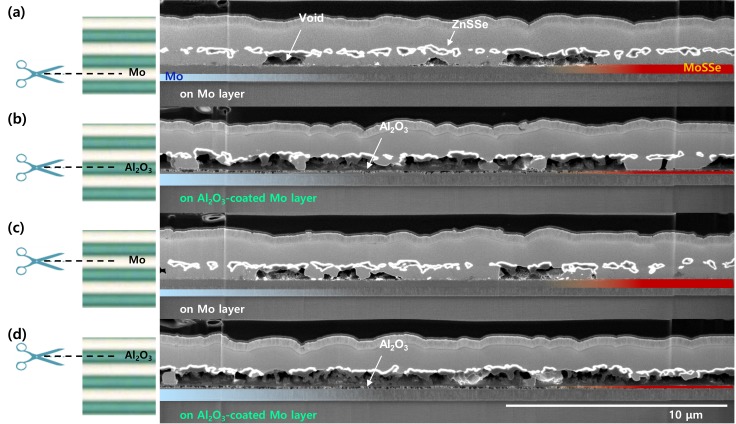
Cross-sectional FESEM images of the self-aligned CZTSSe film on the (**a**,**c**) Mo layer (without the Al_2_O_3_ line pattern) and (**b**,**d**) with the Al_2_O_3_ line pattern; cross-sectional views were obtained after FIB sampling (Ga ion milling).

To confirm the effect of Al_2_O_3_ patterning on the distribution of the bottom CZTSSe and the MoSSe thickness, FESEM measurements were conducted, with cutting performed along the direction parallel to the Al_2_O_3_ line pattern, as shown in Figure 2. Figure 2a,c shows a cross-section of the Mo-exposed area, and thick MoSSe and a relatively small portion of voids are observed. Figure 2b,d. shows a cross-section of the Al_2_O_3_-coated area, and thin MoSSe and a relatively large portion of voids are observed. Thus, we can confirm that a high portion of voids form in the region where Al_2_O_3_ is coated, and a high portion of bottom CZTSSe is present in the regions where Mo is exposed.

**Figure 3:**
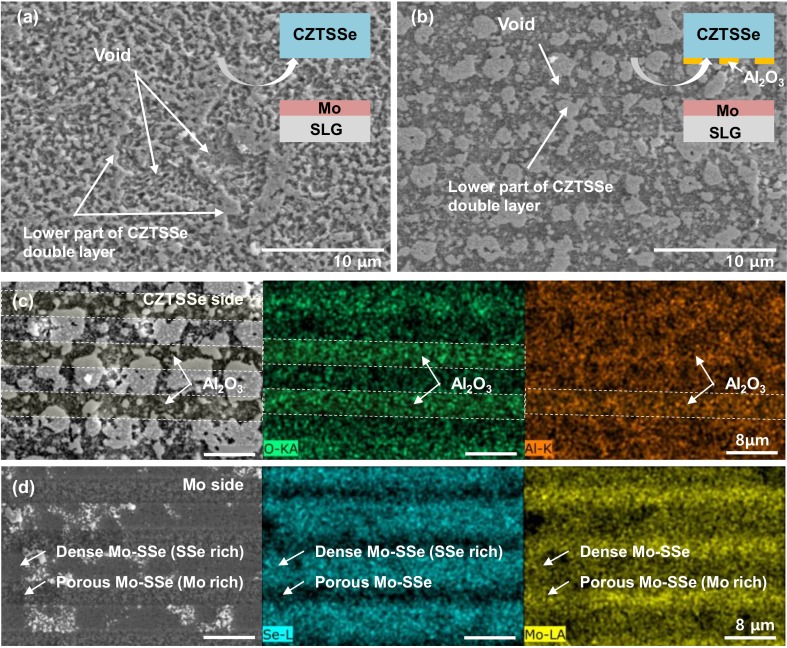
Surface FESEM images of the CZTSSe side after exfoliation for the (**a**) Mo-SLG and (**b**) Al_2_O_3_-patterned Mo-SLG substrates. (**c**) Surface FESEM-EDS images (maps for O and Al) of the CZTSSe side after exfoliation for the Al_2_O_3_-patterned Mo-SLG substrate. (**d**) Surface FESEM-EDS images (maps for Se and Mo) of the Mo side after exfoliation for the Al_2_O_3_-patterned Mo-SLG substrate.

To observe the distribution of the bottom CZTSSe, the CZTSSe layer was exfoliated, and the surface morphology of the exfoliated CZTSSe side is shown in Figure 3.

Figure 3a shows the case of using a Mo-SLG substrate without Al_2_O_3_ patterning. A random distribution of the bottom CZTSSe and various sizes of voids are observed. Figure 3b shows FESEM images of the exfoliated CZTSSe on the Al_2_O_3_-patterned Mo-SLG substrate. Most Al_2_O_3_ is expected to be attached to the CZTSSe side, as shown in Figure 3c. Due to the poor adhesion with MoSSe. The bottom CZTSSe is relatively agglomerated and aligned along the exposed lines of the Mo electrode. As shown in Figure 3d, dense MoSSe forms at the positions where Al_2_O_3_ is not coated, and a relatively porous MoSSe forms at the positions where Al_2_O_3_ is coated. Although the Al_2_O_3_ layer coated by sputtering does not completely block the formation of MoSSe, it is effective.

**Figure 4:**
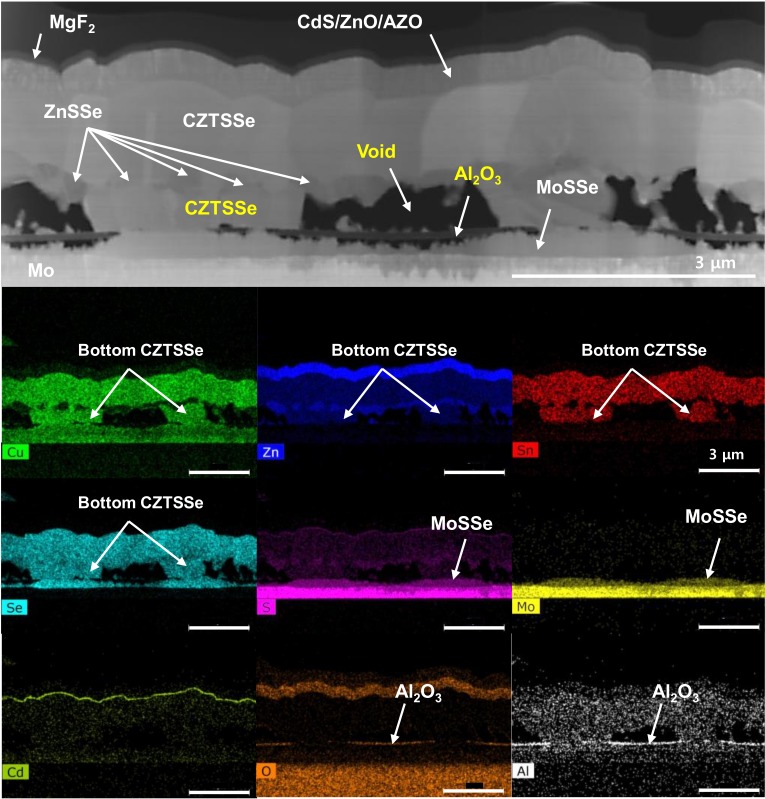
Cross-sectional STEM-EDS images (maps for Cu, Zn, Sn, Se, S, Mo, Cd, O and Al) of CZTSSe on the Al_2_O_3_-patterned Mo-SLG substrate.

Figure 4 shows a cross-sectional STEM-EDS mapping image of self-aligned CZTSSe. The CZTSSe double layer, ZnSSe layer, and void distribution are shown in Figure 4. As mentioned above, a dense CZTSSe layer is found in the top CZTSSe layer, and a discontinuous ZnSSe layer is observed between the top and bottom CZTSSe layers. The Al_2_O_3_ layer is confirmed by the mapping images of Al and O, and the CZTSSe of the lower part is confirmed to have self-assembled at the locations where Mo is exposed. Thick MoSSe is observed at the Mo-exposed positions, and thin MoSSe is found under the Al_2_O_3_ film.

Thus far, the void formation mechanism has been understood based on the decomposition model shown in Equation (1) [9,10]: CZTSSe can be decomposed by Mo, volatile SnSSe readily forms, and a void develops by volatilization of the SnSSe phase at the Mo interface region [10].

2Cu_2_ZnSnS(e)_4_ + Mo → 2Cu_2_S(e) + 2ZnS(e) + 2SnS(e) + MoS(e) (1)

However, based on our results, the decomposition model cannot be applied when using a metal precursor. The self-alignment phenomenon of the bottom CZTSSe double layer is expected to occur due to the difference in the wetting properties between the liquid metal and the substrate. As described in our previous work [7], the η-Cu_6_Sn_5_ and ε-Cu_3_Sn phases formed under the ZnSSe layer, which was preferentially formed from the Sn/Cu/Zn metal precursor by sulfo-selenization. Liquid Sn is believed to be produced under the ZnSSe layer when the process temperature is increased through the peritectic reaction point (408 °C, η-Cu_6_Sn_5_ → ε-Cu_3_Sn + liquid Sn) [7,11]. Additionally, the liquid Cu-Se phase might diffuse near to the Cu-Sn alloys (ε-Cu_3_Sn + liquid Sn) and form the Cu-Sn-Se phase under the ZnSSe layer [7,12], and finally, the bottom CZTSSe might be formed by the reaction between the Cu-Sn-SSe and ZnSSe layers. Thus, the origin of the self-alignment phenomenon of the bottom CZTSSe is expected to be based in the different wetting properties between liquid Sn and the different surfaces, such as the Mo-exposed area and the Al_2_O_3_-coated area.

**Figure 5:**
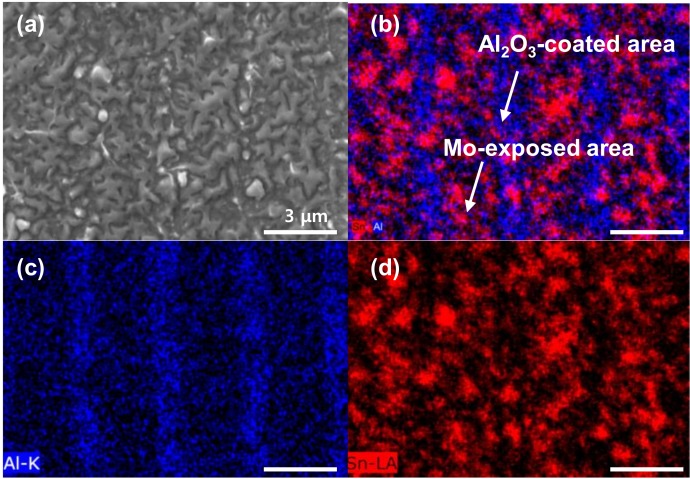
Surface (**a**) FESEM and (**b**) FESEM-EDS image (maps for Al and Sn) of Sn on the Al_2_O_3_-patterned Mo-SLG substrate; Al is shown in blue, and Sn is shown in red. FESEM-EDS mapping of (**c**) Al and (**d**) Sn components. For the wetting test, the Sn film was deposited by sputtering and annealed at 400 °C for 10 min under Ar flowing conditions.

To observe how real liquid Sn is distributed on a substrate having two surfaces of Al_2_O_3_ and Mo at a temperature of approximately 400 °C, Sn was deposited on the Al_2_O_3_-patterned Mo-SLG substrate, which was then annealed at 400 °C for 10 min under Ar flow, as shown in Figure 5. The 400°C-annealed Sn exhibits a typical island shape rather than a continuous film, as shown in Figure 5a. In particular, the mapping analysis reveals that a relatively large amount of Sn is present on the Mo-exposed surfaces compared with that on the Al_2_O_3_-coated surface, which is probably due to the wetting between liquid Sn and Mo being better than that between liquid Sn and Al_2_O_3_ [8].

**Figure 6:**
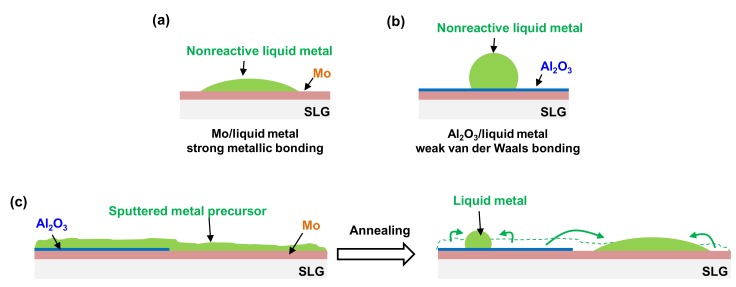
Schematic diagram of the differences in wettability of the (**a**) Mo-SLG substrate and (**b**) Al_2_O_3_-coated Mo-SLG substrate. (**c**) Schematic diagram of the self-alignment mechanism between a liquid metal and different surfaces.

Figure 6 shows a schematic diagram of the wetting characteristics between a nonreactive liquid metal and solid metal. The wettability between the liquid metal and solid metal is generally good due to the strong metallic bonding [13]. However, the wettability between a nonreactive liquid metal and an ionocovalent oxide layer is generally poor due to the weak van der Waals bonding between the liquid metal and the oxide layer [13], as shown in Figure 6b. When liquid Sn is reflowed over the Al_2_O_3_ layer, it moves relatively easily because the wetting is poor. However, when liquid Sn is wetted at a position where Mo is exposed, the movement of liquid Sn is expected to be hindered because the wetting is good. As a result, liquid Sn can be collected at a position where Mo is exposed, as shown in Figure 6c.

**Figure 7:**
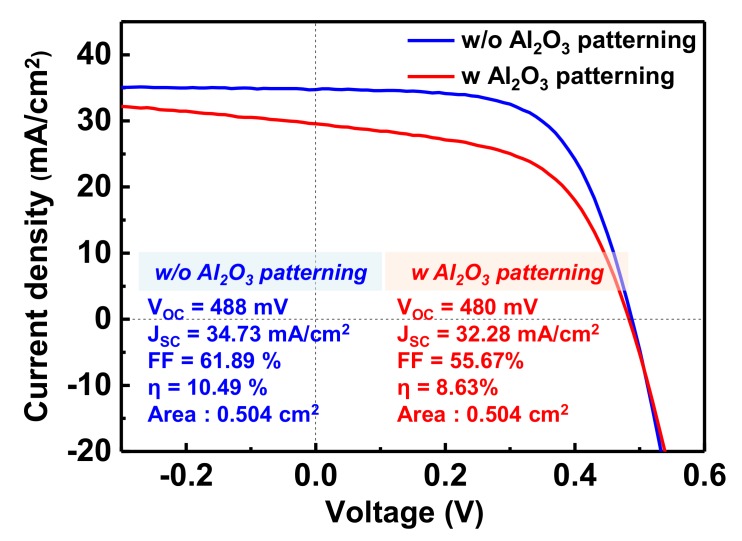
J-V curves of the CZTSSe devices without an antireflection layer.

As a result, the efficiency of the CZTSSe-aligned device without an antireflection layer is 8.6%, as shown in Figure 7. which is lower than that of the device without Al_2_O_3_ patterning. Interestingly, the open-circuit voltages (V*_OC_*) are similar, but the short-circuit current (J*_SC_*) and F.F. (fill factor) values of the device with Al_2_O_3_ are relatively low. Furthermore, the electrical parameters were determined by fitting J-V curves according to the one-diode model described by Hegehus and Shafarman [14]. The deduced data show a slight increase in the series resistance (R*_S_*) from 0.99 Ω cm^2^ (without Al_2_O_3_ patterning) to 1.09 Ω cm^2^ (with Al_2_O_3_ patterning) and a decrease in the shunt resistance (R*_sh_*) from 775.2 Ω cm^2^ (without Al_2_O_3_ patterning) to 134.6 Ω cm^2^ (with Al_2_O_3_ patterning). This result indicates that the resistance of the back contact of the cell does not increase due to the patterned Al_2_O_3_ thin-film layer. The decrease in the F.F. with the decrease in R*_sh_* may occur for various reasons. For example, since voids are not a closed pore state but rather an open pore state, they seem to be able to act as a shunt path, as a contamination pathway in the CBD process, or as an external moisture transfer pathway. Additionally, if the composition of the absorber layer is not optimized, then the generation of the secondary phase leads to a shunt path due to the high conductivity, resulting in F.F. degradation [15,16].

When the cause of the self-alignment of voids and the bottom CZTSSe at the Mo back contact region is taken as the wettability between the liquid metal and the substrate surface, a way to enhance the power conversion efficiency can be suggested as follows. First, the composition of the metal precursor should be optimized using an intermediate-layer-patterned Mo/SLG substrate. Second, another intermediate layer can be applied; carbon materials (C) and ionocovalent ceramic (e.g., BN) substrates are candidate materials due to their poor wettability of liquid metals [13]. Third, the thickness and film quality of the intermediate layer should be modified to completely inhibit MoSSe formation. Fourth, the parameter of patterning should be optimized; patterning for open points should be design based on the diffusion length which derived carrier lifetime in the light absorption layer [17].

## 4. Conclusions

Herein, we report that the Al_2_O_3_ intermediate layer can control the position of void when the ununiform void formation is inevitable; when CZTSSe is formed using an Sn/Cu/Zn stacked metal precursor, ununiform void formation is inevitable. The CZTSSe of the lower part self-aligns on the Mo-exposed area, while the voids self-align on the Al_2_O_3_-coated area. The origin of the self-alignment is expected to be the difference in the wetting characteristics between the liquid metal and the different substrate surfaces. When using a Mo substrate over which a patterned oxide exists to realize local contact passivation, the above phenomenon should be considered, especially in the pure metal precursor case. The efficiency of the CZTSSe-aligned device without an antireflection layer is 8.6%, which is lower than that of the device without Al_2_O_3_ patterning. Nevertheless, our results show that we can effectively control the void or bottom CZTSSe distribution. In general, Al_2_O_3_ interlayers are applied to realize interfacial defect passivation effects and passivated emitter and rear cell (PERC) effects in the field of CIGS thin-film solar cells [17]. Thus, in the process of forming a high-quality CZTSSe absorbing layer using a metal precursor, when the Al_2_O_3_ intermediate layer is used to realize the interfacial defect passivation effect and PERC effect, the results of this experiment should be further considered. In future work, other materials will be applied as an intermediate layer using different coating techniques to increase the carrier transport properties and inhibit MoSSe formation. Also, we will investigate the effect of interface passivation and PERC using optimized patterning.
